# Prevalence, predictors and consequences of gambling on Children in Ghana

**DOI:** 10.1186/s12889-022-14750-0

**Published:** 2022-12-02

**Authors:** Sylvester Kyei-Gyamfi, Daniel Coffie, Michael Ofori Abiaw, Patience Hayford, Joseph Otchere Martey, Frank Kyei-Arthur

**Affiliations:** 1 Department of Children, Ministry of Gender, Children and Social Protection, Accra, Ghana; 2 Department of Environment and Public Health, University of Environment and Sustainable Development, Somanya, Ghana

**Keywords:** Gambling, Prevalence, Predictors, Consequences, Children, Ghana

## Abstract

**Background:**

Gambling is illegal for children in Ghana. However, young people, including children, are increasingly engaged in gambling across the country. Gambling is harmful and poses developmental implications for the youth, including children. There are limited studies on child gambling in Ghana and other sub-Saharan African countries. This study examined the prevalence of gambling participation, predictors of gambling participation, and perceived harm of gambling on children.

**Methods:**

A cross-sectional convergent parallel mixed methodology was used to study 5024 children aged 8 to 17 in the three ecological zones of Ghana. Binary logistic regression was used to examine the predictors of gambling participation while the qualitative data was analysed thematically.

**Results:**

Overall, 3.1% of children aged 8 to 17 had ever engaged in gambling activities. Also, being a female (AOR = 0.210, Wald test = 34.413, 95% CI: 0.125–0.354), having attained primary education (AOR = 4.089, Wald test = 18.643, 95% CI: 2.158–7.749), doing paid work (AOR = 2.536, Wald test = 10.687, 95% CI: 1.452–4.430), having a friend who gambles (AOR = 1.587, Wald test = 4.343; 95% CI: 1.028–2.451), having access to radio (AOR = 1.658, Wald test = 6.694, 95% CI: 1.130–2.431) and having access to mobile phone (AOR = 0.590, Wald test = 6.737, 95% CI: 0.396–0.879) were predictors of gambling participation. Gambling is perceived as harmful by children since it is addictive, affects learning and class attendance, and makes them steal from their families to gamble.

**Conclusions:**

This study demonstrated that socio-demographic characteristics (sex, age, educational attainment, ecological zone, persons taking care of children, living arrangement, engagement in paid work, radio access, mobile phone access) and gambling-related information (having friends who gamble) make children more susceptible to gambling. Researchers and policymakers should consider these socio-demographic characteristics and gambling-related information when designing interventions to curb gambling among children aged 8 to 17 in Ghana.

**Supplementary Information:**

The online version contains supplementary material available at 10.1186/s12889-022-14750-0.

## Background

Since its inception in 1960, the gambling industry in Ghana has served as a source of revenue for the government. The gambling industry supports the government through payments into the country’s Consolidated Fund, donations to the poor and vulnerable through its corporate social responsibility activities, payment of taxes and job creation. For instance, the National Lottery Authority contributed GH₵ 16 million Ghana Cedis and GH₵ 30 Ghana Cedis to the country’s Consolidated Fund in 2016 and 2017, respectively [1].

Currently, gambling operations in the country are managed and controlled by the Gaming Commission of Ghana (GCG) through the Gaming Act, 2006 (Act 721) [2]. The GCG is responsible for issuing licenses to all gaming operators and permits to companies and individuals that wish to run promotions with games of chance. Gaming activities regulated by the GCG include casinos, sports betting, slot machines, and promotions [3]. In Ghana, gambling is legal for adults but illegal for persons under 18. According to Sect. 48 of Act 721, “a person responsible for a gambling machine shall not permit a child to use the gambling machine or to enter a place where the gambling machine is operated” [2]. However, it is worth noting that while gambling is illegal for persons under 18, there is no agency or institution strictly monitoring or imposing this law.

There is evidence of increasing engagement of young people, including children, in sports betting and other gambling activities in the country. For example, a study by Odame et al. [4] found that young people, including children, engaged in sports betting, card games, poker machines, and lotteries in the country. Similarly, Zagoon-Sayeed [5] found that children aged 10 – 17 residing in Tamale, Kumasi, Takoradi and Accra were engaged in sports betting. In recent times, the Ghanaian media has also reported an increasing incidence and engagement of young people in gambling across the country [6]. According to Ghanasoccernet [7], sports betting in Ghana has exponentially risen, especially among youth. Similarly, Nyavor [8] reported the proliferation of online sports betting via mobile gadgets among Ghanaian youth. The author attributed the expansion of online sports betting to increased penetration of mobile phones, mobile money and the internet.

Empirical studies have established that gambling is harmful and poses developmental implications for the youth, including children. For example, engagement in gambling activities is associated with an increased risk of child abuse or neglect [9], depression and anxiety [10], substance abuse [10], suicidal behaviour [11, 12], disruption to academics and social lives [10], stealing from family [10].

Despite these negative impacts of gambling on youth, including children, there are limited studies on the adverse effects of gambling in Ghana and other sub-Saharan African countries [13]. For example, in Ghana, limited studies on adolescent gambling have focused on problem gambling and its predictors [4], participation and attitudes towards gambling [14], perceptions about automated slot/poker machines [15], and initiation, understanding and perceived benefits of gambling [16]. However, these studies were not representative of the entire country. Thus, there is insufficient understanding of the gambling situation among children aged 8 to 17 in Ghana and its adverse effects. Comprehensive knowledge of child gambling would help policymakers and researchers design appropriate interventions to tackle children’s engagement in gambling in the country. As such, this study examined the prevalence of gambling participation, predictors of gambling participation, and perceived harm of gambling on children.

## Methods

### Study design and sampling procedure

This study used a convergent parallel mixed methodology to examine gambling among children aged 8 to 17. A convergent parallel mixed method enables researchers to concurrently collect quantitative and qualitative data to understand a phenomenon [17]. With the convergent parallel mixed method, the researchers place equal importance on the quantitative and qualitative data. The data are analysed separately but interpreted together.

In this study, researchers employed a systematic sampling procedure. First, researchers selected 20 percent of districts in each of the then ten regions of Ghana based on child protection issues such as child labour, child marriage, and general wellbeing of children. In total, 43 districts were chosen from 216 districts across the country. Second, researchers selected 15 enumeration areas from each of the 43 districts. In total, 645 enumeration areas were selected. Third, researchers used probability proportional to size sampling to select children aged 8 to 17 from each district. In total, 5,024 children aged 8 to 17 were interviewed for the quantitative data. Finally, ten focus group discussions (FGDs) were conducted with children aged 8 to 17 for the qualitative data in 10 communities across the country. Each FGD had a maximum of 14 members.

### Study setting

Ghana is a West African country with a 30.8 million population as of 2021. Ghana shares a boundary with Burkina Faso in the North, the Gulf of Guinea in the South, Togo in the East and Cote d’Ivoire in the West. In addition, Ghana has about 42 percent of its population aged below 18. There is a higher proportion of males (43%) than females (41%) aged below 18 years [18]. As of 2018, Ghana had ten regions. However, the number of regions increased from ten to sixteen regions after four regions were split into ten regions after a referendum held on December 27, 2018 [19].

### Description and conceptualisation of variables

#### Dependent variable

The dependent variable for this study is gambling participation, which is an indicator variable (Yes/No) of whether children had ever gambled. This measurement of gambling participation is similar to a previous study in Nigeria [20], which measured gambling participation by ever gambled.

#### Independent variables

The independent variables for this study included socio-demographic characteristics and gambling-related information. The socio-demographic characteristics of children included sex (male and female), age (8 to 10 years, 11 to 13 years, and 14 to 17 years), educational attainment (not in school, primary, Junior High School (JHS), and Senior High School (SHS) and higher), living arrangement (both biological parents, biological mother alone, biological father alone, and other), religion (Christianity, Islam, and other), whether respondent had their own children (Yes and No), person caring for respondent (Father, Mother, Self, and Other), doing any paid work (Yes and No), consider oneself as physically healthy (Yes and No), being a happy person (Yes, No, Some of the Time, and Don’t Know), access to a radio (Yes and No), access to a television (Yes and No), access to a mobile phone (Yes and No), access to the internet (Yes and No), and access to a computer (Yes and No). Also, ecological zone was recategorised as Coastal zone (Western, Central, Greater Accra and Volta regions), Middle belt (Eastern, Ashanti and Brong Ahafo regions) and Northern zone (Northern, Upper East and Upper West).

In addition, gambling-related information, such as having friends who gamble and perceiving sports betting as a game or gambling, were included as independent variables. Having friends who gamble was categorised as Yes and No, while perceiving sports betting as a game or gambling was classified as Gambling, Game and Don’t know.

### Data collection

This study is part of a larger study on the situation of children residing in Ghana. The situational analysis examined several topics, including but not limited to the quality, accessibility, and availability of education, health, water, sanitation and social welfare facilities and services available to children; and the benefits of educational, health, water, sanitation and social welfare facilities and services on children at the community level.

Data collection was carried out between April and October 2018. A day seminar was organised for field assistants across the country to train them on the data collection instruments (semi-structured questionnaire and interview guides for key informant interviews and FGDs). The trained field assistants interviewed children aged 8 to 17, adults, and workers of District Assemblies and their respective departments dealing with child-related issues. The data collection instruments covered similar topics, including background information on children, their household and living conditions, education, health, deviant behaviour (alcohol abuse, abortion, gambling, etc.), and other related information necessary for policymaking. However, this study focused on gambling-related issues among children aged 8 to 17.

The data collected for this study is part of the monitoring and evaluation of the Ministry of Gender, Children and Social Protection (MoGCSP) across the country. The Institutional Review Board of the Department of Children, MoGCSP reviewed and approved this study. Children aged 8 to 17 who agreed to participate in the study signed the written informed consent form; parents or legal guardians signed the written informed consent form of all children aged 8 to 17. The data collection adhered to the principles of the declaration of Helsinki. In addition, children's rights were safeguarded, and the data were anonymised to protect children's privacy.

### Data analysis

The quantitative data was analysed using the Statistical Package for the Social Sciences (SPSS) version 26. The socio-demographic characteristics of children and gambling-related information were described using descriptive statistics. In addition, Pearson’s chi-square and Fisher’s exact tests were used in examining the association between socio-demographic characteristics, gambling-related information and gambling participation. Also, binary logistic regression was used to determine the predictors of gambling participation.

The researchers assessed the data to ensure the assumptions of binary logistic regression were met (such as specification error, multicollinearity, independence of observations, and linearity). For instance, the researchers carried out linktest command in STATA to ensure all relevant variables were included in the binary logistic regression (See Table 1 in additional file). The linear predicted value (_hat) was statistically significant (*p*-value = 0.000), while the linear predicted value squared (_hatsq) was not statistically significant (*p*-value = 0.121). This means the model is properly specified and all relevant variables have been included in the model. Also, the researchers confirmed that all variables included in the model were not highly correlated (See Table 2 in additional file). The variance inflation factor (VIF) value for each variable was less than 2. Each observation in the dataset was unique, and there were no outliers.

Two models were fitted for this study. Model I examined the relationship between all the socio-demographic characteristics and gambling participation. Model II examined gambling-related information in addition to the socio-demographic characteristics and their relationship with gambling participation. Adjusted Odds Ratio, a type of odds ratio that controls for other independent variables in a model, will be reported in the results section and Models I and II.

All qualitative data were audio recorded and transcribed verbatim. Field notes and transcripts were analysed thematically. The authors read all transcripts several times to get a general sense of gambling among children. The authors assigned codes to statements that provided relevant information about the research objectives. The authors grouped similar codes into themes and sub-themes. To ensure trustworthiness, the authors analysed the data together, and disagreements on themes and sub-themes were resolved. In addition, the authors consulted peers experienced in qualitative study design and analysis during the data collection and analysis.

## Results

### Socio-demographic characteristics of respondents

Table 1 shows the socio-demographic characteristics of respondents. More than half (51.1%) of respondents were male, and a higher proportion of males (5.3%) had participated in gambling than females (0.8%) (*p*-value = 0.000). Less than half of respondents were aged 14 to 17 (43.7%), had attained primary education (48.5%) and resided in the Middle belt (40.7%). A higher proportion of respondents aged 14 to 17 (6.2%) had participated in gambling than those aged 8 to 10 (0.4%) and 11 to 13 (1.0%).

**Table 1:** Socio-demographic characteristics of respondents

Variables		Gambling participation		
	Frequency (%)	No Frequency (%)	Yes Frequency (%)	*p*-value
Sex				
Male	2566 (51.1)	2429 (94.7)	137 (5.3)	86.995***
Female	2458 (48.9)	2439 (99.2)	19 (0.8)	
Age				
8 – 10	1330 (26.5)	1325 (99.6)	5 (0.4)	124.437***
11 – 13	1497 (29.8)	1482 (99.0)	15 (1.0)	
14 – 17	1607 (43.7)	2061 (93.8)	136 (6.2)	
Mean age	12.9			
Standard deviation	3.0			
Educational attainment				
Not in School	813 (16.2)	789 (97.0)	24 (3.0)	17.320**
Primary	2436 (48.5)	2382 (97.8)	54 (2.2)	
JHS	1496 (29.8)	1433 (95.8)	63 (4.2)	
SHS and higher	279 (5.5)	264 (94.6)	15 (5.4)	
Ecological zone				
Coastal zone	1806 (35.9)	1707 (94.5)	99 (5.5)	55.653***
Middle belt	2043 (40.7)	1999 (97.8)	44 (2.2)	
Northern zone	1175 (23.4)	1162 (98.9)	15 (1.1)	
Living arrangement				
Both biological parents	2883 (57.4)	2812 (97.5)	71 (2.5)	22.546***
Biological mother alone	987 (19.6)	958 (97.1)	29 (2.9)	
Biological father alone	225 (4.5)	208 (92.4)	17 (7.6)	
Other	929 (18.5)	890 (95.8)	39 (4.2)	
Religion				
Christianity	3766 (75.0)	3649 (96.9)	117 (3.1)	3.145
Islam	1088 (21.7)	1058 (97.2)	30 (2.8)	
Other	170 (3.4)	161 (94.7)	9 (5.3)	
Whether respondent had their own children				
Yes	107 (2.1)	106 (99.1)	1 (0.9)	1.712^&^
No	4917 (97.9)	4762 (96.8)	155 (3.2)	
Person caring for respondent				
Father	250 (5.0)	248 (99.2)	2 (0.8)	15.213^& **^
Mother	853 (17.0)	839 (98.4)	14 (1.6)	
Self	3467 (69.0)	3348 (96.6)	119 (3.4)	
Other	454 (9.0)	433 (95.4)	21 (4.6)	
Doing any paid work				
Yes	200 (4.0)	176 (88.0)	24 (12.0)	54.775***
No	4824 (96.0)	4692 (97.3)	132 (2.7)	
Consider oneself as physically healthy				
Yes	3753 (74.7)	3619 (96.4)	134 (3.6)	10.679**
No	1271(25.3)	1249 (98.3)	22 (1.7)	
Being a happy person				
Yes	3430 (68.3)	3321 (96.8)	109 (3.2)	17.862***
No	289 (5.8)	270 (93.4)	19 (6.6)	
Some of the time	575 (11.4)	558 (97.0)	17 (3.0)	
Don’t know	730 (14.5)	719 (98.5)	11 (1.5)	
Access to radio				
Yes	2904 (57.8)	2797 (96.3)	107 (3.7)	7.681**
No	2120 (42.2)	2071 (97.7)	49 (2.3)	
Access to television				
Yes	3472 (69.1)	3361 (96.8)	111 (3.2)	0.316
No	1552 (30.9)	1507 (97.1)	45 (2.9)	
Access to mobile phone				
Yes	1770 (35.2)	1715 (96.9)	55 (3.1)	0.000
No	3254 (64.8)	3153 (96.9)	101 (3.1)	
Access to internet				
Yes	336 (6.7)	316 (94.0)	20 (6.0)	9.703**
No	4688 (93.3)	4552 (97.1)	136 (2.9)	
Access to computer				
Yes	453 (9.0)	438 (96.7)	15 (3.3)	0.070
No	4571 (91.0)	4430 (96.9)	141 (3.1)	

^&^ = Fisher’s Exact Test value; *p* < 1 ∗ , *p* < 0*.*05 ∗  ∗ , *p* < 0*.*01 ∗  ∗  ∗

Regarding educational level, respondents with SHS and higher education reported a higher prevalence (5.4%) of gambling participation. A higher proportion of respondents residing in the Coastal zone (5.5) had participated in gambling compared with those living in the Middle belt (2.2%) and Northern zone (1.1%). In addition, the majority of respondents lived with their biological parents (57.4%), were Christians (75%), had no children (97.9%), and took care of themselves (69%). A higher proportion of respondents who lived with only their biological fathers (7.6%) had participated in gambling.

Most respondents cared for themselves (69%), did not engage in any paid work (96%) and perceived themselves as physically healthy (74.7%). A higher proportion of respondents who were cared for by others (4.6%), who engaged in paid work (12%), and who were physically healthy (3.6%) had participated in gambling.

Most respondents (68.3%) perceived themselves as happy, and 3.2% of those who perceived themselves as happy (*p*-value = 0.000) had participated in gambling. In terms of access to electronic gadgets, most respondents had access to a radio (57.8%) and television (69.1%). A higher proportion of respondents with access to a radio (3.7%) had participated in gambling than those who had no access to a radio (2.3%). However, the majority of respondents had no access to a mobile phone (64.8%), computer (91.0%) or internet (93.3%). Respondents who had internet access reported a higher prevalence (6.0%) of participated in gambling than those without internet access (2.9%).

### Gambling-related information

Table 2 shows that most respondents had no friends who gambled (91%) and perceived football betting as gambling (83.5%). A higher proportion of respondents who had a friend who gambled (10.3%) had participated in gambling. Likewise, respondents who perceived football betting as gambling reported a higher prevalence (4.5%) of gambling participation.

**Table 2:** Gambling-related information

Variables		Gambling participation		
	Frequency (%)	No Frequency (%)	Yes Frequency (%)	*p*-value
Have friends who gamble				
Yes	438 (8.7)	393 (89.7)	45 (10.3)	81.963***
No	4586 (91.3)	4475 (97.6)	111 (2.4)	
Perceive football betting as a game or gambling				
Gambling	3264 (83.5)	3118 (95.5)	146 (4.5)	12.717^&^ **
Game	248 (6.4)	242 (97.6)	6 (2.4)	
Don’t know	395 (10.1)	391 (99.0)	4 (1.0)	

^&^ = Fisher’s Exact Test value; *p* < 1 ∗ , *p* < 0*.*05 ∗  ∗ , *p* < 0*.*01 ∗  ∗  ∗

### Prevalence of gambling participation

In this study, 3 out of 100 children aged 8 to 17 reported that they had participated in gambling (Table 3). Among those who gambled, the majority (81.4%) participated in sports bets. Also, most respondents (77.8%) indicated they knew what gambling is.

**Table 3:** Information on gambling among children aged 8 to 17

Variables	Frequency	Percent
Have you ever gambled		
Yes	156	3.1
No	4868	96.9
Types of gambling engaged in		
Sports bet	127	81.4
Card games	5	3.2
Slot games/poker	24	15.4
Know what gambling is		
Yes	3907	77.8
No	1117	22.2

### Predictors of gambling participation

Table 4 shows the predictors of gambling participation. Model II, the final model, revealed that female children (AOR = 0.210; Wald test = 34.413; 95% CI = 0.125–0.354; *p*-value = 0.000), children aged 8 to 10 (AOR = 0.032; Wald test = 44.069; 95% CI = 0.012–0.088; *p*-value = 0.000), children aged 11 to 13 (AOR = 0.080; Wald test = 64.095; 95% CI = 0.043–0.149; *p*-value = 0.000), Middle belt (AOR = 0.493; Wald test = 12.235; 95% CI = 0.331–0.733; *p*-value = 0.000) and Northern zone (AOR = 0.204; Wald test = 21.514; 95% CI = 0.104–0.399; *p*-value = 0.000) were less likely to have participated in gambling.

**Table 4:** Binary logistic regression of predictors of gambling participation

Variables	Model I			Model II		
	AOR	Wald	95% CI	AOR	Wald	95% CI
Sex						
Male (RC)						
Female	0.135 ∗ ∗ ∗	60.934	0.082–0.223	0.210 ∗ ∗ ∗	34.413	0.125–0.354
Age		96.660			89.171	
8 – 10	0.030 ∗ ∗ ∗	50.713	0.011–0.079	0.032 ∗ ∗ ∗	44.069	0.012–0.088
11 – 13	0.080 ∗ ∗ ∗	65.448	0.044–0.148	0.080 ∗ ∗ ∗	64.095	0.043–0.149
14 – 17 (RC)						
Educational attainment		21.200			21.705	
Not in School (RC)						
Primary	3.931 ∗ ∗ ∗	18.461	2.105–7.339	4.089 ∗ ∗ ∗	18.643	2.158–7.749
JHS	1.704 ∗	3.430	0.969–2.996	1.712 ∗	3.356	0.963–3.045
SHS and higher	1.801	2.388	0.854–3.797	1.743	2.061	0.816–3.721
Ecological zone		37.337			27.701	
Coastal zone (RC)						
Middle belt	0.442 ∗ ∗ ∗	16.906	0.299–0.652	0.493 ∗ ∗ ∗	12.235	0.331–0.733
Northern zone	0.171 ∗ ∗ ∗	27.829	0.089–0.330	0.204 ∗ ∗ ∗	21.514	0.104–0.399
Living arrangement		6.494			7.658	
Both biological parents (RC)						
Biological mother alone	0.963	0.024	0.597–1.555	0.951	0.041	0.586–1.544
Biological father alone	2.202 ∗ ∗	5.941	1.167–4.155	2.388 ∗ ∗	6.958	1.251–4.559
Other	1.077	0.090	0.665–1.744	1.060	0.055	0.650–1.729
Religion		5.793			7.057	
Christianity	0.587	1.684	0.263–1.312	0.615	1.328	0.269–1.405
Islam	1.006	0.000	0.419–2.415	1.154	0.098	0.469–2.841
Other (RC)						
Whether respondent had their own children						
Yes	0.393	0.803	0.051–3.034	0.396	0.783	0.051–3.087
No (RC)						
Person caring for respondent		12.876			10.394	
Father (RC)						
Mother	3.246	2.217	0.689–15.286	2.812	1.695	0.593–13.337
Self	5.464 ∗ ∗	5.242	1.277–23.379	4.403 ∗ ∗	4.007	1.031–18.793
Other	9.594 ∗ ∗	8.403	2.080–44.252	7.648 ∗ ∗	6.779	1.654–35.375
Doing any paid work						
Yes	2.454 ∗ ∗	10.663	1.432–4.206	2.536 ∗ ∗	10.687	1.452–4.430
No (RC)						
Consider oneself as physically healthy						
Yes	1.570 ∗	3.103	0.950–2.593	1.451	2.091	0.876–2.404
No (RC)						
Being a happy person		8.036			6.390	
Yes (RC)						
No	1.925 ∗ ∗	4.652	1.062–3.492	1.772 ∗	3.478	0.971–3.231
Some of the time	0.670	1.871	0.377–1.189	0.678	1.730	0.380–1.210
Don’t know	0.952	0.020	0.480–1.887	1.074	0.041	0.539–2.138
Access to radio						
Yes	1.586 ∗ ∗	5.736	1.062–3.492	1.658 ∗ ∗	6.694	1.130–2.431
No (RC)						
Access to television						
Yes	0.913	0.197	0.612–1.363	0.868	0.468	0.579–1.302
No (RC)						
Access to mobile phone						
Yes	0.591 ∗ ∗	6.777	0.398–0.878	0.590 ∗ ∗	6.737	0.396–0.879
No (RC)						
Access to internet						
Yes	1.530	1.846	0.829–2.823	1.515	1.673	0.807–2.844
No (RC)						
Access to computer						
Yes	0.812	0.397	0.425–1.551	0.810	0.396	0.420–1.562
No (RC)						
Have friends who gamble						
Yes				1.587 ∗ ∗	4.343	1.028–2.451
No (RC)						
Perceive football betting as a game or gambling					0.278	
Gambling (RC)						
Game				1.187	0.120	0.450–3.134
Don’t know				0.815	0.138	0.278–2.394

*AOR *Adjusted Odds Ratio, *RC *Reference category; *p* < 1 ∗ , *p* < 0.05 ∗  ∗ , *p* < 0.01 ∗  ∗  ∗

Conversely, primary education (AOR = 4.089; Wald test = 18.643; 95% CI = 2.158–7.749; *p*-value = 0.000), living with biological father alone (AOR = 2.388; Wald test = 6.958; 95% CI = 1.251–4.559; *p*-value = 0.008), children caring for themselves (AOR = 4.403; Wald test = 4.007; 95% CI = 1.031–18.793; *p*-value = 0.045), others being responsible for children (AOR = 7.648; Wald test = 6.779; 95% CI = 1.654–35.375; *p*-value = 0.009), children engaged in paid work (AOR = 2.536; Wald test = 10.687; 95% CI = 1.452–4.430; *p*-value = 0.001), children who had access to radio (AOR = 1.658; Wald test = 6.694; 95% CI = 1.130–2.431, *p*-value = 0.010), children who had access to mobile phone (AOR = 0.590; Wald test = 6.737; 95% CI = 0.396–0.879, *p*-value = 0.009), and children who had friends who gambled (AOR = 1.587; Wald test = 4.343; 95% CI = 1.028–2.451, *p*-value = 0.037) were more likely to have participated in gambling.

### Perceived harm of gambling to children

Children aged 8 to 17 were asked their views regarding the harmful effects of gambling. Four themes emerged from the analysis of transcripts: gambling is addictive, children sometimes steal from their families to gamble, gambling affects learning, and gambling affects school attendance.

### Gambling is addictive

Respondents explained that gambling could lead to gambling addiction. Children engaged in gambling are sometimes addicted to it and always find money to gamble after unsuccessful attempts. Respondents elaborated that gambling (sports betting) should be done occasionally rather than continuous since it is a game of chance.

*Some children use their pocket money for sports betting; even when they lose, they find money and wager again. I believe sports betting is a game of chance, so you do not have to do it all the time. You try it occasionally when there is a major match. Incidentally, many of my friends believe they will win in their next attempt, which usually ends again in failure. (FGD, Coastal Zone)*

### Children sometimes steal from their families to gamble

Some respondents expressed that gambling makes children steal from their families. With neither a livelihood nor any other source of money, many child gamblers find it challenging to finance their gambling activities. Consequently, some children use illegal means, including stealing, to finance their gambling activities.

*Sometimes you need money to wager on a match you believe a team has a strong chance of winning. Very often, most children do not have money to do it. Most children become desperate and are compelled to borrow or steal to place a wager. There have been instances when I have stolen from my mother and younger siblings to bet. (FGD; Coastal Zone)*

### Gambling affects learning

Some respondents indicated that gambling makes child gamblers depressed when they lose, which negatively affects their learning and, subsequently, examination performance. One respondent shared his experience in the following statement:

*On the eve of my term exams, I bet on a match between Chelsea and Real Madrid, expecting Chelsea to win. Unfortunately, Chelsea lost to Real Madrid, and I felt so sad I could not prepare well for my paper the next day. I failed the paper because I was still thinking about the money I lost throughout the exams. (FGD, Middle Belt)*

### Gambling affects school attendance

Some respondents highlighted that gambling keeps children out of school. Respondents narrated that some children go to gambling facilities, such as pubs, during school contact hours. A respondent illustrated it in the statement below:

*Some children go to pubs to play roulette, and poker whilst class is ongoing in school. There have been instances when the District Assembly officials have engaged in swoops and seized slot/poker machines in such places. (FGD, Coastal Zone)*

## Discussion

This study investigated the prevalence of gambling participation, predictors of gambling participation, and perceived harm of gambling on children. The study found that only three hundredths (3.1%) of children aged 8 to 17 had ever engaged in gambling. The prevalence of gambling among children in this study is lower than the prevalence found in other studies. For instance, Glozah, Tolchard and Pevalin’s [14] study among 770 Ghanaian Senior High School students found that 21.1% engaged in sports betting, 4.5% engaged in card games, 2.9% engaged in poker machines, while 1.7% engaged in lotteries. In Nigeria, Aguocha et al.’s [20] study among 507 male secondary school students found that the majority (77.6%) of respondents had gambled in the last 12 months. In the United States, Hurt et al.’s [21] study among 384 adolescents aged 10–12 found that 27.2% of respondents had participated in gambling for money. Another study in the United States by GRAPC and UNICEF [22] among 1000 adolescent aged 14–17 found that 13% of respondent had ever gambled.

Among the previous studies, only Aguocha’s [20] study conducted in Nigeria used a similar measurement of child gambling participation. Glozah, Tolchard and Pevalin’s [14] study in Ghana measured youth gambling participation using four gambling types (lotteries, card games, sports betting, and poker machines). Also, GRAPC and UNICEF’s study in the United States measured child gambling participation using seven gambling types (online casino page, casino, hand play, slot game, sports game, lottery, and any game for money) [22]. Another study in the United States [21] measured child gambling participation by whether children had ever gambled for money by playing card game, sports betting or lottery. The difference in the measurement of child gambling participation in previous studies could also contribute to differences in the prevalence of gambling participation among children.

The study also found that sex, age, educational attainment, ecological zone of residence, living arrangement, person taking care of respondent, engagement in paid work, radio access, mobile phone access and having friends who gambled were significant predictors of gambling participation. Males were more likely to have participated in gambling than females. This finding corroborates other studies that found that males are more likely to engage in gambling activities than females. For example, a study by Hurt et al. [21] among 384 preadolescents aged 10–12 in the United States found that males had higher odds of being gamblers than females. Similarly, Forrest and McHale’s [23] study among children aged 11–15 in Britain found that males were more likely to gamble than females. Generally, males are more likely to take financial risks than females [24], which may explain the increased odds of gambling participation.

Older children (14 to 17 years) were more likely to have participated in gambling than younger children (8 to 13 years). This finding confirms previous studies that children's engagement in gambling activities increases with age [25]. Conversely, other studies found that engagement in gambling activities reduces with age. For instance, Forrest and McHale’s [23] study among children in Britain found that older children are less likely to experience problem gambling than younger children. Similarly, Odame’s [4] study among adolescents in rural Ghana found that older adolescents are less likely to experience problem gambling than younger adolescents. According to Aflakpui and Oteng-Abayie [26], gambling activities, especially sports betting, are more attractive to younger persons than older persons. Discrepancies in findings on age and gambling participation could be attributed to differences in the study populations and instruments used to measure gambling participation.

Regarding educational attainment, respondents who were not in school had reduced odds of gambling participation than those who are in school. A plausible explanation is that education makes it easier for children to access and understand information on gambling which may increase their participation in gambling activities. Also, the findings revealed that respondents who resided in the Coastal zone were more likely to have participated in gambling than those in the Middle belt and Northern zones. A probable explanation is that there is a proliferation of various types of gambling, such as sports betting and slot/poker machines, in Greater Accra, located in the Coastal zone, than in other parts of the country. According to Adogla-Bessa [27], there is proliferation of sports betting in resource-limited urban areas in Accra.

Children living with their biological father alone had increased odds of gambling participation than children who lived with both biological parents. This finding highlights that living with both biological parents is a protective factor against engagement in gambling activities. In addition, living with both biological parents provides better parental supervision than living with either a mother or father.

Also, children who took care of themselves and those taken care of by others were more likely to have participated in gambling than those who were taken care of by their fathers. This finding emphasises that when fathers are responsible for taking care of their children, they serve as a protective factor against participation in gambling activities than when children take care of themselves and when taken care by others, including extended family members. Children who were engaged in paid work were more likely to have participated in gambling than those who were not involved in any paid work. The income earned from paid work may have given those children the financial means to participate in gambling activities. Forrest and McHale’s [23] study found that children with more money were more likely to gamble than their counterparts with less money.

Furthermore, children who were not happy were more likely to have participated in gambling than those who were happy. This finding is similar to a study in the United States of America that found that children participate in gambling activities when they are sad [22].

Concerning radio access, children who had radio access were more likely to have participated in gambling than those without radio access. Radio is one of the main mediums used by betting companies, especially sports betting, to market their products. The increased exposure of younger persons, including children, to gambling advertisements makes them perceive gambling activities as acceptable, harmless and fun [28, 29], making them more likely to participate in gambling activities. For instance, a systematic review by Killick and Griffiths [30] found that increased exposure to sports betting advertisement increases participation in sports betting. The authors also reported that individuals are exposed to sports betting advertisements via social media, radio, and billboards. They further highlighted that sports betting advertisements are inevitable during live telecast of betting sports events.

Regarding access to mobile phones, children with access to mobile phones had increased odds of gambling participation than those without access to mobile phones. Increased access to mobile phones has removed the physical barrier to gambling activities, especially sports betting. All sports betting companies in Ghana have online applications which are easy to access via smart mobile phones. Sports betting companies also allow bettors to deposit and withdraw money from their betting accounts via Mobile Money. These developments may explain why children with access to mobile phones are more likely to have participated in gambling than those without access to mobile phones. According to Ghana Business News [31], four-fifths (80%) of Ghanaian sports bettors use mobile gadgets to stake their bets.

Furthermore, children whose friends gambled are more likely to have participated in gambling. This finding highlights the influence of friends on gambling behaviour. This finding supports previous studies that found that the participation of friends in gambling activities negatively influenced their peers/colleagues to participate in those activities [21, 32–34].

Moreover, children mentioned that gambling is harmful. They explained that gambling is addictive and sometimes makes children steal from their families to gamble. In addition, gambling affects children learning when they lose, and it also affects their class attendance. For instance, children gamblers explained that when they lose a gamble, they look for more money to wager again with the hope of recovering their losses. According to Cusack et al. [35], gamblers are often pressured to gamble again when they lose with the hope of recovering their losses.

Some children gamblers indicated that they had to steal from their families to finance their gambling escapades. In addition, previous studies have documented that when gamblers often lose money, they are sometimes compelled to engage in illegal activities, such as stealing, to wager again [35, 36].

The study also highlighted that when children gamblers sometimes lose their bets, they become anxious and depressed, which negatively affects their academic performance. In addition, studies have found an association between depression symptoms and gambling on the one hand, and poor academic performance and gambling on the other [37].

Finally, the study reported that gambling made some children gamblers miss class since they preferred to spend their time at gambling facilities rather than in the classroom. Previous studies have established that children who engage in gambling often spend much time at gambling facilities, making them miss their classes [14, 22].

## Strength and limitations of the study

This study has some limitations. First, this study used a single question (whether children had ever gambled) to measure gambling participation, which is not robust. Second, children were asked if they had participated in gambling. Since gambling is illegal for persons below 18 years, children may underreport their gambling experiences. Also, there may be recall bias since children had to reminisce about their gambling experience, which may lead to underreporting their experiences. Fourth, this study is cross-sectional, so we cannot infer causal relationships between the dependent and independent variables. Fifth, there were more males who had participated in gambling than females. In addition, there were more children aged 14 to 17 than children aged 8 to 10 and 11 to 13. This makes the sample non-representative of the overall population. Six, previous studies have documented essential variables that influence child gambling participation. These include but are not limited to ethnicity, parental gambling attitudes and behaviour (including parent gambling frequency), parental marital status, child marital status, child alcohol and tobacco use, perceived social difficulties from family, friends, and school, and sexual abuse victimisation [4, 14, 23]. However, the authors could not include them in the Models due to data limitations. The omission or non-inclusion of such vital variables can affect the reliability of the estimates obtained in this study. Despite these limitations, the national representative nature of the data would enable researchers and policymakers to comprehensively understand the gambling phenomenon and its associated predictors among children aged 8 to 17 to design appropriate interventions to deal with gambling among children in Ghana.

## Conclusion

This study found that gambling participation was less prevalent among children aged 8 to 17 than in previous studies in Ghana, Nigeria, Britain, and the United States. In addition, this study demonstrated that socio-demographic characteristics (such as being male, older age, educational attainment, residing in coastal zones, engagement in paid work, living with only a biological father, caring for oneself, being cared for by others, radio access, and mobile phone access) and gamble-related information (such as having friends who gamble) make children more susceptible to gambling. The study also revealed that gambling is harmful to children since it is addictive, makes children steal from families to gamble, and affects learning and class attendance. Researchers and policymakers should consider these socio-demographic characteristics and gambling-related information when designing interventions to curb gambling among children aged 8 to 17 in Ghana.

## Supplementary Information

**Additional file 1:** Supplementary file1

## Data Availability

The datasets generated and/or analysed during the current study are not publicly available due to them containing information that could compromise research participant privacy but are available from the corresponding author on reasonable request.

## Abbreviations

AOR: Adjusted Odds Ratio
FGDs: Focus Group Discussions
GCG: Gaming Commission of Ghana
JHS: Junior High School
MoGCSP: Minsitry of Gender, Children and Social Protection
RC: Reference Category
SHS: Senior High School
