# Supplementary material for: Prevalence, predictors and consequences of gambling on Children in Ghana
Source: BMC Public Health. 2022 Dec 2;22:2248. doi: 10.1186/s12889-022-14750-0 (PMC9719180; doi:10.1186/s12889-022-14750-0)
Supplement: Supplementary file 1 — Additional file 1: Supplementary file1 [file 12889_2022_14750_MOESM1_ESM.docx]

**Table 1: Results of linklest**

| **linktest** | | | | | | |
| --- | --- | --- | --- | --- | --- | --- |
| Iteration 0: log likelihood = -695.20417  Iteration 1: log likelihood = -551.38713  Iteration 2: log likelihood = -510.61818  Iteration 3: log likelihood = -505.5925  Iteration 4: log likelihood = -504.98322  Iteration 5: log likelihood = -504.97597  Iteration 6: log likelihood = -504.97597 | | | | | | |
| Logistic regression Number of obs = 5,024 | | | | | | |
| LR chi2(2) = 380.46 | | | | | | |
| Prob > chi2 = 0.0000 | | | | | | |
| Log likelihood = -504.97597 Pseudo R2 = 0.2736 | | | | | | |
| Ever_Gambled_RECODE_NEW | Coef. | Std. Err. | z | P>\|z\| | [95% Conf. Interval] | |
| _hat | 1.256733 | .1827973 | 6.88 | 0.000 | .8984566 | 1.615009 |
| _hatsq | .0438937 | .0283335 | 1.55 | 0.121 | -.011639 | .0994264 |
| _cons | .2758761 | .2547901 | 1.08 | 0.279 | -.2235033 | .7752554 |

**Table 2: Results of multicollinearity test**

| **Coefficients** | | | | | | | |
| --- | --- | --- | --- | --- | --- | --- | --- |
| **Model** | **Unstandardised Coefficients** | | **Standardised Coefficients** |  |  | **Collinearity Statistics** | |
|  | **B** | **Std. Error** | **Beta** | **t** | **Sig.** | **Tolerance** | **VIF** |
| (Constant) | 0.129 | 0.068 |  | 1.914 | 0.056 |  |  |
| Sex | -0.047 | 0.006 | -0.118 | -7.350 | 0.000 | 0.924 | 1.083 |
| Age | 0.037 | 0.005 | 0.155 | 8.253 | 0.000 | 0.672 | 1.488 |
| Educational attainment | 0.004 | 0.004 | 0.015 | 0.856 | 0.392 | 0.799 | 1.251 |
| Ecological zone | -0.025 | 0.005 | -0.095 | -5.544 | 0.000 | 0.813 | 1.231 |
| Living arrangement | 0.002 | 0.003 | 0.013 | 0.791 | 0.429 | 0.856 | 1.168 |
| Religion | 0.019 | 0.006 | 0.049 | 2.984 | 0.003 | 0.868 | 1.152 |
| Respondent had their own children | 0.042 | 0.023 | 0.030 | 1.857 | 0.063 | 0.914 | 1.094 |
| Person caring for respondent | 0.015 | 0.005 | 0.048 | 2.951 | 0.003 | 0.886 | 1.128 |
| Doing any paid work | -0.073 | 0.016 | -0.073 | -4.560 | 0.000 | 0.919 | 1.088 |
| Consider oneself as Physically healthy | -0.007 | 0.007 | -0.016 | -0.980 | 0.327 | 0.926 | 1.080 |
| Being a happy person | 1.910E-06 | 0.000 | 0.000 | 0.020 | 0.984 | 0.908 | 1.101 |
| Access to radio | -0.014 | 0.006 | -0.036 | -2.326 | 0.020 | 0.975 | 1.025 |
| Access to TV | 0.002 | 0.007 | 0.005 | 0.309 | 0.757 | 0.913 | 1.096 |
| Access to mobile phone | 0.022 | 0.007 | 0.054 | 3.263 | 0.001 | 0.874 | 1.144 |
| Access to internet | -0.010 | 0.014 | -0.013 | -0.731 | 0.465 | 0.801 | 1.248 |
| Access to computer | 0.013 | 0.011 | 0.020 | 1.155 | 0.248 | 0.836 | 1.197 |
| Have friends who gamble | -0.044 | 0.011 | -0.066 | -4.047 | 0.000 | 0.885 | 1.130 |
| Perceive football betting as a game or gambling | 0.006 | 0.005 | 0.020 | 1.210 | 0.226 | 0.838 | 1.193 |
